# Barriers to childhood immunisation: Findings from the Longitudinal Study of Australian Children

**DOI:** 10.1016/j.vaccine.2015.04.089

**Published:** 2015-06-26

**Authors:** Anna Pearce, Helen Marshall, Helen Bedford, John Lynch

**Affiliations:** a Population, Policy and Practice, UCL Institute of Child Health, University College London, London, United Kingdom; b School of Population Health, University of Adelaide, South Australia, Australia; c Robinson Research Institute and School of Paediatrics and Reproductive Health, University of Adelaide, South Australia, Australia; d Vaccinology and Immunology Research Trials Unit, Women's and Children's Hospital, South Australia, Australia; e School of Social and Community Medicine, University of Bristol, Bristol, United Kingdom

**Keywords:** Infants, Vaccination, Health inequalities, Social disadvantage, Population health

## Abstract

**Objectives:**

To examine barriers to childhood immunisation experienced by parents in Australia.

**Design:**

Cross-sectional analysis of secondary data.

**Setting:**

Nationally representative Longitudinal Study of Australian Children (LSAC).

**Participants:**

Five thousand one hundred seven infants aged 3–19 months in 2004.

**Main outcome measure:**

Maternal report of immunisation status: incompletely or fully immunised.

**Results:**

Overall, 9.3% (473) of infants were incompletely immunised; of these just 16% had mothers who disagreed with immunisation. Remaining analyses focussed on infants whose mother did not disagree with immunisation (*N* = 4994) (of whom 8% [398] were incompletely immunised).

Fifteen variables representing potential immunisation barriers and facilitators were available in LSAC; these were entered into a latent class model to identify distinct clusters (or ‘classes’) of barriers experienced by families. Five classes were identified: (1) ‘minimal barriers’, (2) ‘lone parent, mobile families with good support’, (3) ‘low social contact and service information; psychological distress’, (4) ‘larger families, not using formal childcare’, (5) ‘child health issues/concerns’. Compared to infants from families experiencing minimal barriers, all other barrier classes had a higher risk of incomplete immunisation. For example, the adjusted risk ratio (RR) for incomplete immunisation was 1.51 (95% confidence interval: 1.08–2.10) among those characterised by ‘low social contact and service information; psychological distress’, and 2.47 (1.87–3.25) among ‘larger families, not using formal childcare’.

**Conclusions:**

Using the most recent data available for examining these issues in Australia, we found that the majority of incompletely immunised infants (in 2004) did not have a mother who disagreed with immunisation. Barriers to immunisation are heterogeneous, suggesting a need for tailored interventions.

## Highlights

• A known group of under-immunising parents in Australia are conscientious objectors.
• However we found that most under-immunising parents do not disagree with immunisation.
• These parents experience a heterogeneous range of barriers to immunisation.
• Eg low social contact, psychological distress, large families, child health concerns.
• Tailored interventions are required to address these varying needs.

## 1 Introduction

The ‘Immunisation Australia: Seven Point Plan’ [1] was implemented in 1997, with the aim of increasing uptake of childhood immunisations. Strategies introduced included school entry immunisation requirements and a series of financial incentives for parents and general practitioners [1–3]. Coverage increased rapidly, from an estimated 53% in the 1980s [4] to over 90% (in 12–15 month old infants) by 2000 [5]. However, uptake has since remained relatively stable at around 91–92% [5], which is below the Organisation for Economic Cooperation and Development average [6]; and in some social groups and areas of Australia coverage does not reach the level required for herd immunity [7]. Consequently, the 2013–2018 National Immunisation Strategy aims to increase immunisation coverage further, and ensure equity of access regardless of financial and geographic barriers [4].

Two broad groups of non-immunising parents are described in the literature [8]. The first are ‘conscientious objectors’ or hesitant parents with concerns about immunisation who may decline, delay or be selective in the vaccines they accept; these parents tend to be more affluent and educated [9–11]. The second group comprises families experiencing barriers to access, which may relate to social disadvantage and logistical barriers [11–13]. Interventions to increase uptake in these two groups require different approaches. Conscientious objection has increased over recent years, and today tens of thousands of conscientious objections are recorded on the Australian Childhood Immunisation Register (ACIR) each year [14]. This has prompted the investigation of the societal and cultural influences on vaccine acceptance (e.g. media, public health policies, and moral or religious beliefs) [9], and the development of a framework for health professionals to address parental concerns [8]. However, it is possible that between half [11] and 80% of children who are not fully immunised do not have a parent who conscientiously objects to immunisation (according to ACIR data, in 2012 8% of 12–15 month olds were not fully immunised, and 1.5% of all registered children had a conscientious objection recorded [14]). No study has examined individual-level barriers such parents may experience and socio-economic inequalities in immunisation since implementation of the Seven Point Plan in 1997. Using nationally representative data from a cohort of infants born 6 years after the introduction of Australia's 1997 Seven Point Plan [1], we examined the potential barriers to immunisation experienced by parents who did not disagree with immunisation.

## 2 Methods

### 2.1 Participants

Children registered on the Medicare database were selected into the Longitudinal Study of Australian Children (LSAC) using a stratified cluster sample to ensure proportionate geographic representation of the states/territories and those residing within and outside capital state statistical divisions [15]. Interviews were carried out by trained interviewers in the home, with the primary caregiver (the mother in 98.6% of cases, thus referred to as mother hereafter). The study protocol was approved by the Australian Institute of Family Studies Ethics Committee.

Our analysis focussed on children in born in 2003–2004; 8921 were invited to participate and 5107 (57%) recruited. The first survey was carried out when the children were aged 3–19 months. Further information on LSAC is available elsewhere [16].

### 2.2 Measures

#### 2.2.1 Immunisation status

Mothers were asked:

• ‘Overall how much do you agree with children being immunised, that is having their needles or injections (five-point Likert scale from very strongly agree to very strongly disagree, and don’t know)’.
• ‘Is *child's name* up to date with his/her immunisations, that is needles or injections? (yes completely up to date; no but has had most; no but has had some; no hasn’t had any; don’t know)’.

To aid their answers throughout the survey, the majority (91.6%) used the Child Health and Development Record (‘Baby Book’), which (if complete) contained information on immunisations received. Infants were classified as being fully immunised or incompletely immunised (received most, some or no immunisations). Mothers were categorised as disagreeing or not disagreeing with childhood immunisation.

#### 2.2.2 Barriers to immunisation

**Fig. 1:**
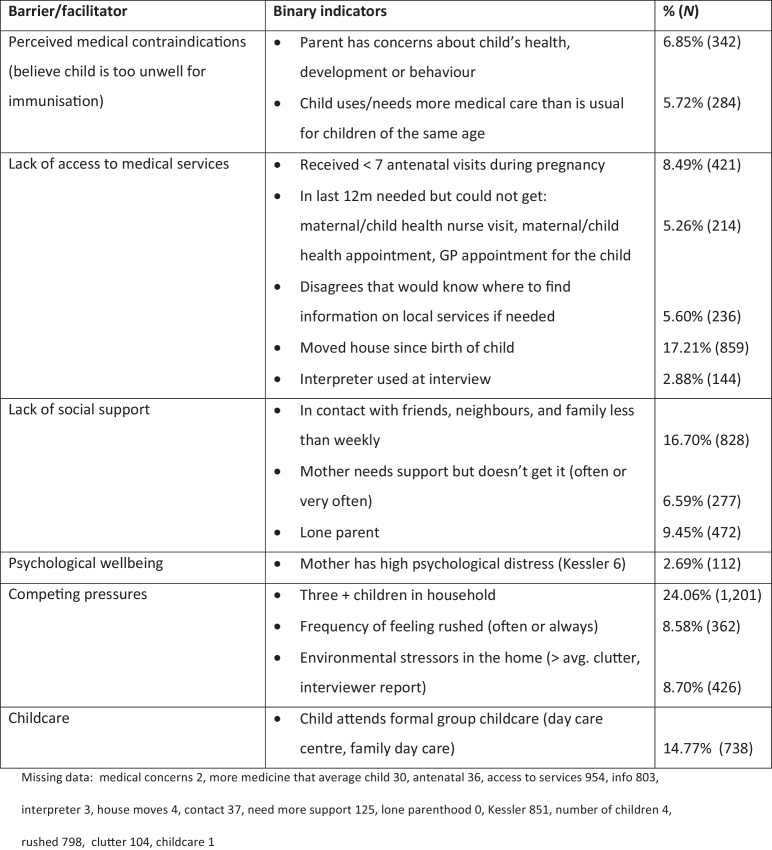
Indicators of barriers and facilitators to immunisation in mothers who did not disagree with immunisation: description and prevalence (*N* = 4994).

Fifteen indicators of potential barriers/facilitators were available (Fig. 1), representing: perceived medical contraindications (parents may believe that their child is too ill to be immunised [there are few, extremely rare, genuine contraindications [17]]), lack of access to medical services, lack of social support, maternal psychological well being, competing pressures (such as large families) and formal group childcare (at the time of data collection, parents were eligible for childcare assistance if their child was fully immunised [2], and childcare providers may encourage or require children to be immunised). These variables were dichotomised and entered into a latent class model to identify clusters of barriers within the population (see Section 2.3).

#### 2.2.3 Demographic and socio-economic characteristics

We examined a number of demographic characteristics and indicators of parental socioeconomic position: the child's age (in months), gender, Aboriginal and Torres Strait Islander status, whether one or more parents were born in Australia, quintiles of area disadvantage (Socio-Economic Indexes for Areas), remoteness of residence, mother's highest level of education, and weekly household income.

We adjusted for these variables as confounders of the association between the exposure (barriers to immunisation) and outcome (immunisation). Additionally, we describe the prevalence of incomplete immunisation according to these characteristics.

### 2.3 Statistical analysis

#### 2.3.1 Descriptive statistics

We estimated the overall prevalence of immunisation (accounting for the sample design). We then examined uptake according to parental disagreement towards immunisation. Subsequent analyses were carried out only in infants whose mother did not disagree with immunisation (*N* = 4994).

#### 2.3.2 Creating a measure of potential barriers to immunisation

We used latent class analysis (LCA) to characterise families experiencing different clusters (or ‘classes’) of barriers, according to the 15 indicators (Fig. 1). Two sets of parameters were estimated: ‘class membership probabilities’ (the relative size of the classes) and ‘item response probabilities’ (the probability of children in a given class experiencing each of the 15 barrier indicators) [18]. Models ranging up to seven classes were considered (as providing a useful reduction of the data), and the following taken into account when selecting the final model: Akaike information criterion (AIC), Bayesian information criterion (BIC), class posterior probabilities (likelihood of members of an assigned class truly belonging to that class), and entropy (the precision of membership assignment across all individuals) [18]. We also considered interpretability, that is, the extent to which each class was distinct from the others (in terms of the barrier indicators experienced). A five-class model was identified as the most parsimonious (see Appendix 1). Children were assigned to the class that they had the highest probability of belonging to [18]. Analyses were carried out using a Stata plug-in [19] for the SAS procedure PROC LCA [18], accounting for sample weights.

#### 2.3.3 Examining the effect of potential barriers to immunisation on immunisation uptake

Poisson regression was used to estimate unadjusted and adjusted risk ratios (RRs) and 95% confidence intervals (CIs) for incomplete immunisation, according to the five barrier classes identified in the LCA. We estimated RRs before and after adjustment for potential confounding by demographic and socio-economic characteristics. Analyses were carried out in Stata 13.0 (StataCorp, College Station, TX).

### 2.4 Sensitivity analyses

The robustness of the five-class barrier measure was considered through repeating the LCA in three ways: (1) excluding mothers who ‘neither agreed nor disagreed’ with immunisation, (2) without adjustment for sample weights, and (3) including the outcome (immunisation status) in the latent class model. A five-class model remained appropriate in all cases, and the types of barriers experienced by the different classes were very similar (data not shown).

We identified the five-class barrier as the most appropriate measure based on a number of factors described earlier. However, selection of a latent class measure is always subjective. We therefore repeated our final analytical model, using the three- and seven-class measures (which also had relatively good model fits) as the exposure variable. Use of either of these did not change the overall conclusions (see Appendix 1).

The final adjusted regression model was repeated excluding the 8% of mothers who did not use the ‘Baby Book’ to aid their answers during the survey, and also excluding children who were completely unimmunised (to check that associations were not substantially different to those who were partially immunised). In both cases the results were very similar and are not reported.

The immunisation question was designed to capture current status regardless of age (which ranged from 3 to 19 months). However younger infants had less time to catch-up, and older infants were due additional vaccines such as the measles, mumps and rubella vaccine (MMR), which is recommended at 12 months [17]. The majority (65%) of infants were aged 7–11 months, and so should have completed their primary immunisations (administered at age 2, 4 and 6 months) but not yet any others. The final adjusted regression model was repeated including only these infants (see Section 3).

### 2.5 Missing data

Almost all (99.9%, *N* = 5100) mothers reported their infant's immunisation status and whether they disagreed with childhood immunisation. Main analyses focussed on the 4994 infants with immunisation data and whose mother did not disagree with immunisation; of these 77.4% (3864) had complete data on all 15 barrier indicators, and all infants had information on at least one indicator. Missingness for each indicator is presented under Fig. 1. The LCA procedure [19] was carried out under the assumption that any systematic difference between the missing and observed values could be explained by the observed data (‘missing at random’ (MAR) [20]); therefore all infants were assigned a barrier class. Three of the eight confounding variables (parents’ country of birth, maternal education, and household income) were missing data, with income missing the most (*N* = 267). These data were imputed, using multiple imputation by chained equations, in twenty datasets, under a MAR assumption. The imputation model included immunisation status, all socio-economic and demographic variables, and the barrier classes. Level of missingness for the variables, and the socio-economic and demographic distribution of the complete and imputed samples, are presented in Appendix 2.

## 3 Results

### 3.1 Immunisation uptake

Table 1 shows that 90.7% of infants were fully immunised; 9.3% were incompletely immunised (of whom 18% had received no immunisations, data not shown). 2.1% (106) mothers disagreed with immunisation either quite or very strongly. Importantly the majority (70.8%) of infants whose mother disagreed with immunisation were incompletely immunised. However, just 15.9% of incompletely immunised infants had a mother who disagreed with immunisation, implying that there were other barriers to immunisation aside from conscientious objection.

Amongst mothers who did not disagree with immunisation, 8% of infants were not fully immunised (of these, just 6.8% (27) had received no immunisations, data not shown). Subsequent analyses focussed on infants whose mothers did not disagree with immunisation (*N* = 4994).

### 3.2 Potential barriers to immunisation

The most prevalent of the 15 barrier indicators were larger families (three+ children) (24.1%), moving house since the birth of the cohort child (17.2%), less than weekly contact with friends and family (16.7%), and use of formal group childcare (14.8%) (Fig. 1). The least prevalent were psychological distress (2.7%) and use of an interpreter during the survey (2.9%). Table 2 presents the item probabilities (the probability of individuals in a given class experiencing each of the barrier indicators) for the five classes of barriers identified in the LCA. Labels were assigned to the classes based on class homogeneity (where individuals in a given class have a high probability of experiencing particular indicators) or class separation (where individuals in a given class have a high or low probability of experiencing particular indicators, relative to other classes). The labels were: (1) ‘minimal barriers’, (2) ‘lone parent, mobile families with good support’, (3) ‘low social contact and service information; psychological distress’, (4) ‘larger families, not using formal childcare’, and (5) ‘child health issues/concerns’. Almost three quarters of children were assigned to the ‘minimal barriers’ class (72%), and the smallest classes were ‘lone parent, mobile families with good support’ (5.4%) and ‘child health issues/concerns’ (4.5%).

### 3.3 Determinants of incomplete immunisation status

In the unadjusted analysis, children whose parents had less education and income were more likely to be incompletely immunised (Table 3). There was no discernible pattern according to area disadvantage, and differences were small.

All barrier classes had a higher risk of incomplete immunisation than infants living in families experiencing ‘minimal barriers’. The extent of the relative difference (represented by RRs) ranged from 1.74 (95% CI: 1.27–2.40) for ‘low social contact and service information; psychological distress’, to 2.81 (2.16–3.67) for ‘larger families, not using formal childcare’. RRs were only slightly attenuated after adjustment for confounders: RR 1.51 (1.08–2.10) in ‘low social contact and service information; psychological distress’ and 2.47 (1.87–3.25) in ‘larger families, not using formal childcare’. When analyses were limited to infants aged 7–11 months, the prevalence of incomplete immunisation was slightly lower at 6.5%. All barriers continued to carry an elevated risk of incomplete immunisation after adjustment for confounders, although the patterns changed slightly (Table 3, final column); most notably, the RR in ‘child health issues/concerns’ was substantially attenuated (1.26 [0.61–2.58]).

## 4 Discussion

Using the most contemporary Australian data available, we have shown that the majority of incompletely immunised infants (in 2004) did not have a parent who disagreed with immunisation, and socioeconomically disadvantaged parents were more likely to be incompletely immunised. Several clusters (or ‘classes’) of barriers were experienced by parents who did not disagree with immunisation: (1) ‘minimal barriers’, (2) ‘lone parent, mobile families with good support’, (3) ‘low social contact and service information; psychological distress’, (4) ‘larger families, not using formal childcare’, and (5) ‘child health issues/concerns’. Compared to ‘minimal barriers’, all other barrier classes displayed an elevated risk of incomplete immunisation; this remained after adjustment for confounding, including individual- and area-level socioeconomic disadvantage.

This is the first Australia-wide study showing that factors relating to socioeconomic disadvantage (such as low maternal education [11,13]) identified in earlier research continue to be associated with immunisation uptake after the introduction of the Seven Point Plan. Recent national reports using ACIR data indicate that immunisation uptake in the most deprived areas is slightly higher than in the most advantaged areas [7]. Our analysis also found few differences according to area deprivation; however large inequalities according to individual-level characteristics, such as maternal education, were observed. This implies that area-level data may be disguising important associations occurring at the family-level. Furthermore, we have demonstrated that a number of barriers influence immunisation status over and above the effects of socioeconomic and demographic characteristics.

LSAC children were sampled from the Medicare database, which at the time included 98% of children by the time they reached age 12 months [16]. However just 57% of those contacted agreed to take part in the survey. Survey and response weights were utilised when estimating immunisation prevalence and creating the latent measure of barriers, although it remains possible that we have under-estimated the prevalence of vaccine-hesitant parents and also the barriers they experience. Immunisation status was reported by the mother, although the majority (91.6%) referred to the ‘Baby Book’ throughout the survey, and the pattern of results was unchanged when limited to these mothers. Our estimation of immunisation coverage is comparable to national figures in 2004 [5] (when the LSAC data were collected), and it has been postulated that disagreement between parental report and health records is low [21] and not socially distributed [22]. It was not possible to determine whether immunisation was timely for fully immunised infants; similarly, under-immunised infants may have subsequently caught up. The five class measure of barriers to immunisation was derived using LCA, which involves an element of subjectivity. However sensitivity analyses were conducted using two alternative measures (with three and seven classes) to ensure that the conclusions would not have changed if we had selected a different measure (which they did not).

Our analyses refer to data collected a decade ago. However LSAC is the most contemporary resource currently available to address these questions. In addition, the barriers experienced by the LSAC parents persist in Australia today [23]; and while we cannot be sure that these barriers continue to have the same impact on immunisation uptake we argue that, with such a large proportion of incompletely immunised infants not having a conscientiously objecting parent [14], this is likely to remain the case. It is possible that new barriers have emerged since the LSAC data were collected, for example efforts to address General Practitioner (GP) shortages in rural Australia have led to improvements in some but not all areas, and 1 in 20 Australian's continue to live in under-serviced areas [24]. Conversely, a number of recent policy changes have the potential to reduce barriers to immunisation into the future; these include the monitoring of immunisation rates by the National Health Performance Authority [7], a new immunisation strategy [4], and Local Health Networks that may help to improve coordination and integration of primary care [25]. However, an awareness of the social barriers experienced at a national and local level is essential if the full potential of these policies is to be realised.

Immunisation is a highly effective public health intervention. However, despite the introduction of a series of policies and incentives in Australia under the 1997 Seven Point Plan, coverage remains suboptimal (particularly in some geographic areas and social groups), with the risk of reduced herd immunity [7]. A known, identifiable and growing group of non-immunising parents are those who conscientiously object, and it is imperative to address their concerns. However, the majority of incompletely immunised infants in LSAC did not have mothers who disagreed with immunisation but were instead experiencing a heterogeneous range of barriers. Dialogue around the importance and safety of immunisation alone is unlikely to be helpful for these families. Only 6.8% of incompletely immunised infants had received no immunisations at all, implying that the majority of families were in contact with health professionals. All interactions with families, whether in primary, secondary or tertiary care, should be treated as potential opportunities to discuss families’ unique needs and consider barriers that could delay immunisation, including previous reactions or negative experiences within the family. This is particularly important for children with chronic conditions (who are often at increased risk of infection as well as the complications of infection) and while frequently reviewed, may be incompletely immunised due to parental and immunisation provider concerns [11]. The characteristics of local populations should be considered when designing programmes to increase uptake. Reminders and rescheduling cancelled appointments [26] may aid uptake in busy families or if a child is sick on the day of appointment, whereas families experiencing reduced access to services or low social support may benefit from interventions which offer immunisation in alternative settings [27].

## Ethics

Ethics approval was not required because the study was an analysis of secondary data.

## Funding

A.P. is funded by a UK Medical Research Council Fellowship (MR/J012351/1). J.L. is supported by an Australia Fellowship from the National Health and Medical Research Council of Australia (570120) and also receives funding from NHMRC Partnership Project Grants APP1056888 and APP1016281. H.M. was funded by a National Health and Medical Research Council Career Development Fellowship No. 1016272. H.B. is funded by Higher Education Funding Council for England (HEFCE).

All authors had full access to all of the data (including statistical reports and tables) in the study

## Figures and Tables

**Table 1:** Immunisation status according to disagreement with immunisation in all infants: row and column percentages (*N* = 5100).

	Col A: fully immunised		Col B: incomplete		Col C: total	
		Col %		Col %		Col %
Row 1: Agree or abstain	*N* = 4596		*N* = 398		*N* = 4994	
Row %	92.03%		7.97%		100%	
		*99.33%*		*84.14%*		*97.92%*
Row 2: Disagree	*N* = 31		*N* = 74		*N* = 106	
Row %	29.25%		70.75%		100%	
		*0.67%*		*15.86%*		*2.08%*
Row 3: Total	*N* = 4627		*N* = 473		*N* = 5100	
Row %	90.73%		9.27%		100%	
		*100%*		*100%*		*100%*

*Note:* Row and column percentages for immunisation status and mother's agreement with immunisation are shown. Row percentages show the prevalence of complete and incomplete immunisation for children whose mother agreed/abstained (row 1) and disagreed (row 2) with immunisation. Row three presents the prevalence of immunisation overall. The column percentages show the proportion of mothers who agreed/abstained or disagreed with immunisation, for children who were fully immunised (column A) and incompletely immunised (column B). Column C shows the proportion of mothers who didn’t and disagree in the entire sample. Missing: disagreement with immunisation (5); immunisation status (3).

**Table 2:** Item probabilities for each of the individual barrier indicators, according to the five barrier classes identified in the latent class analysis (in mothers who did not disagree with immunisation, *N* = 4994).

Class labels:	1. Minimal barriers	2. Lone parent, mobile families with good support	3. Low social contact and service info; psychological distress	4. Larger families, not using formal childcare	5. Child health issues/concerns
Class membership probabilities:	0.68	0.05	0.15	0.08	0.05
Item response probabilities:					
Perceived contraindications					
Concerned about child's health/development/behaviour	0.04	0.10	0.12	0.03	*0.39*
*Child* *>* *average medical needs*	0.02	0.03	0.01	0.03	***0.85***
Access to health services					
<7 antenatal visits	0.06	0.16	0.12	0.21	0.08
Poor access to services	0.03	0.04	0.17	0.05	0.09
1+ moves since birth of child	0.15	**0.51**	0.27	0.07	0.11
Can’t find info on services	0.03	0.06	*0.31*	0.05	0.11
Interpreter used at interview	0.02	0.03	0.10	0.04	0.04
Social support					
Low contact with friends, family etc.	0.08	0.32	**0.52**	0.29	0.20
Need help, but don’t get	0.04	*0.00*	0.26	0.16	0.11
Lone parent family	0.03	***0.97***	0.10	0.20	0.10
Psychological well being					
Psychologically distressed	*0.00*	0.06	*0.19*	0.04	0.07
Competing pressures					
3+ children	0.17	0.26	0.20	***0.98***	0.27
Rushed	0.06	0.02	0.24	0.25	0.15
Cluttered home	0.06	0.25	0.12	0.22	0.09
Childcare					
Formal childcare	0.15	0.17	0.13	*0.02*	0.20
%^a^ children assigned to class	71.94%	5.40%	9.02%	9.17%	4.46%

Item probabilities indicate the likelihood of individuals in a given class displaying each item. Bold highlights indicate class homogeneity (high absolute probabilities [>0.5]), and italic highlights indicate class separation (high relative difference in the item response probabilities [+/−3]).

a Weighted to account for sample design. For measures of model fit, see Appendix 1.

**Table 3:** Unadjusted and adjusted risk ratios (RRs) for incomplete immunisation, according to barrier classes and confounding variables: in infants whose mothers who did not disagree with immunisation: all ages, and limited to ages 7–11 months (final column).

	Infants of all ages (3–19 months), *N* = 4994			7–11 months only, *N* = 3241
	% (*N*)^b^	uRR (95% CI)	aRR^a^ (95% CI)	aRR^a^ (95% CI)
Barrier class				
Minimal barriers	6.10	–	–	–
Lone parent, mobile families, good support	12.86	2.11 (1.45, 3.07)	1.82 (1.18, 2.81)	1.58 (0.87, 2.84)
Low social contact and access to services; psychological distress	10.64	1.74 (1.27, 2.40)	1.51 (1.08, 2.10)	1.87 (1.21, 2.92)
Larger families, not using formal childcare	17.18	2.81 (2.16, 3.67)	2.47 (1.87, 3.25)	2.49 (1.72, 3.62)
Concerns about the child's health’	11.01	1.80 (1.18, 2.75)	1.79 (1.17, 2.73)	1.26 (0.61, 2.58)
Confounding variables				
Area disadvantage				
Most disadvantaged	8.76	1.16 (0.86, 1.57)	0.91 (0.65, 1.27)	1.03 (0.63, 1.71)
Quintile 2	9.04	1.20 (0.88, 1.63)	1.00 (0.72, 1.40)	1.30 (0.79, 2.15)
Quintile 3	8.09	1.07 (0.77, 1.48)	0.96 (0.68, 1.35)	1.26 (0.76, 2.09)
Quintile 4	6.04	0.80 (0.56, 1.14)	0.73 (0.51, 1.05)	0.98 (0.58, 1.68)
Most advantaged	7.55	–	–	–
Remoteness				
Accessible	7.87	–	–	–
Remote	10.43	1.32 (0.86, 2.04)	1.22 (0.78, 1.91)	1.39 (0.79, 2.45)
Unclassified	6.90	0.88 (0.33, 2.35)	0.79 (0.29, 2.15)	1.03 (0.32, 3.32)
Aboriginal and Torres Strait Islander				
No	7.70	–	–	–
Yes	16.15	2.10 (1.41, 3.12)	1.32 (0.85, 2.03)	1.73 (1.03, 2.91)
Parents country of birth				
1+ parents born in Australia	7.96	–	–	–
Neither born in Australia	8.00	1.00 (0.75, 1.33)	1.06 (0.78, 1.42)	0.97 (0.65, 1.46)
Mother's education				
< Year 10	17.36	2.58 (1.74, 3.82)	1.63 (1.04, 2.55)	2.22 (1.22, 4.04)
Year 10–11	9.83	1.46 (1.07, 1.99)	1.14 (0.82, 1.60)	1.47 (0.91, 2.38)
Year 12	7.09	1.05 (0.76, 1.46)	0.96 (0.68, 1.34)	0.87 (0.51, 1.49)
Certificate	8.62	1.28 (0.98, 1.67)	1.11 (0.84, 1.48)	1.62 (1.07, 2.45)
Advanced diploma	5.79	0.86 (0.57, 1.30)	0.81 (0.53, 1.24)	1.10 (0.61, 1.98)
Degree	6.73	–	–	–
Household income				
<$500	10.28	1.57 (1.16, 2.13)	1.00 (0.69, 1.45)	1.22 (0.76, 1.98)
$500–999	9.27	1.42 (1.11, 1.80)	1.20 (0.93, 1.55)	1.35 (0.94, 1.94)
$1000–1999	6.55	–	–	–
$2000+	6.87	1.05 (0.75, 1.47)	1.08 (0.76, 1.55)	1.14 (0.65, 2.00)

a Mutually adjusted for all other measures in the table and child's age (in months) and sex. The association between age and immunisation status was nonlinear, therefore age was categorised as 3–4, 5, 6, 7, 8, 9, 10, 11, 12, 13/19 months and included as a nominal variable.

b Missing data on barriers were imputed during the LCA; missing confounder data were imputed using multiple imputation. See Section 2 for further information.
