# Supplementary material for: Barriers to childhood immunisation: Findings from the Longitudinal Study of Australian Children
Source: Vaccine. 2015 Jun 26;33(29):3377–83. doi: 10.1016/j.vaccine.2015.04.089 (PMC4503793; doi:10.1016/j.vaccine.2015.04.089)
Supplement: Supplementary file 1 [file mmc1.docx]

**Appendix 1: Selection of the latent measure of barrier classes**

The five class model was selected because it had high entropy^, posterior probabilities*, and interpretability (see ‘Statistical analysis’ and Table A1).

*Table A1:*  *Factors used to assess the latent class models*

|  | **1 class** | **2 class** | **3 class** | **4 class** | **5 class** | **6 class** | **7 class** |
| --- | --- | --- | --- | --- | --- | --- | --- |
| *Posterior probabilities*:* | | | | | | | |
|  | 1.00 | 0.84 | 0.84 | 0.82 | 0.87 | 0.71 | 0.86 |
|  |  | 0.77 | 0.71 | 0.69 | 0.70 | 0.72 | 0.74 |
|  |  |  | 0.71 | 0.64 | 0.72 | 0.68 | 0.64 |
|  |  |  |  | 0.68 | 0.58 | 0.65 | 0.72 |
|  |  |  |  |  | 0.74 | 0.80 | 0.72 |
|  |  |  |  |  |  | 0.58 | 0.69 |
|  |  |  |  |  |  |  | 0.61 |
| *Entropy^:* | | | | | | | |
|  | 1 | 0.43 | 0.55 | 0.57 | 0.71 | 0.57 | 0.70 |
| *BIC^^:* | | | | | | | |
|  | 2978 | 2608 | 2600 | 2624 | 2647 | 2726 | 2787 |
| *AIC^^:* | | | | | | | |
|  | 2880 | 2405 | 2293 | 2213 | 2132 | 2106 | 2064 |

*1 indicates perfect assignment within that class; ^1 indicates perfect assignment of all individuals to all classes; *^^*lower values indicate a more parsimonious model

The three class model had the lowest BIC; however entropy was low and class homogeneity and separation was lower than the five class model. The three classes were characterised by 1. ‘Minimal barriers’; 2. ‘Health concerns & medium barriers’; 3. ‘High barriers’. As a sensitivity analysis the final model was repeated using the three class measure in place of the five class one and the ‘Health concerns & medium barriers’ and ‘High barriers’ classes both had an elevated risk of incomplete immunisation compared to ‘Minimal barriers’ (aRR: 1.63 [1.13, 2.36] and 1.95 [1.53, 2.47] respectively).

The seven class model had the lowest AIC, and while entropy was as high as for the five class model, the additional two classes did not increase interpretability, and membership probability of the seventh class was very low (1.6%). The class labels (and aRR for incomplete immunisation) were: 1. ‘Minimal barriers’ (baseline); 2. ‘Lone parent, mobile families with good support’ (1.95 [1.26, 3.01]; 3. ‘Larger families, not using formal (1.40 [0.89, 2.21]); 4. Child health issues/concerns childcare’ (2.04 [0.95, 4.39]); 5. ‘Low social contact and service information’ (1.95 [1.31, 2.90]); 6. ‘Low social contact and support’ (1.36 [0.98, 1.90]); 7. ‘Rushed, distressed and mobile’ (2.47 [1.85, 3.28]).

**Appendix 2: Characteristics of the LSAC sample**

*Table A2: Socio-economic and demographic characteristics of LSAC ‘b-cohort’ in complete and imputed sample (in infants whose mother did not disagree with immunisation)*

|  | **% (*N*) in response sample (*N* varies)** | **% (*N*) in complete sample (*N*=4671)** | **% in imputed sample**  **(*N*=4994)** |
| --- | --- | --- | --- |
| **Area disadvantage, quintiles** |  |  |  |
| Most disadvantaged | 23.77(1187) | 24.02 (1122) | 23.77 |
| Quintile 2 | 20.60 (1029) | 20.68 (966) | 20.60 |
| Quintile 3 | 18.82 (940) | 18.52 (865) | 18.82 |
| Quintile 4 | 18.24 (911) | 18.33 (856) | 18.24 |
| Most advantaged | 18.56 (18.6) | 18.45 (862) | 18.56 |
| *Total* | *100 (4994)* | *100 (4671)* | *100 (4994)* |
| *Missing (N)* | *0* | *323* | *N/A* |
| **Remoteness** |  |  |  |
| Accessible | 94.61 (4725) | 94.75 (4426) | 94.61 |
| Remote | 4.23 (211) | 4.22 (197) | 4.23 |
| Unclassified | 1.16 (58) | 1.03 (48) | 1.16 |
| *Total* | *100 (4994)* | *100 (4671)* | *100 (4994)* |
| *Missing (N)* | *0* | *323* | *N/A* |
| **Aboriginal & Torres Strait** |  |  |  |
| No | 96.78 (4833) | 96.70 (4517) | 96.78 |
| Yes | 3.22 (161) | 3.30 (154) | 3.22 |
| *Total* | *100 (4994)* | *100 (4671)* | *100 (4994)* |
| *Missing (N)* | *0* | *323* | *N/A* |
| **Parents born in Australia?** |  |  |  |
| 1+ parents | 86.74 (4331) | 87.00 (4064) | 86.67 |
| Neither | 13.26 (662) | 13.00 (607) | 13.26 |
| *Total* | *100 (4993)* | *100 (4671)* | *100 (4994)* |
| *Missing (N)* | *1* | *323* | *N/A* |
| **Mother’s education** |  |  |  |
| < year 10 | 3.73 (184) | 3.60 (168) | 3.73 |
| Year 10-11 | 13.17 (650) | 12.89 (602) | 13.16 |
| Year 12 | 15.28 (754) | 15.37 (718) | 15.28 |
| Certificate | 25.03 (1235) | 25.07 (1171) | 25.01 |
| Advanced diploma | 9.73 (480) | 9.76 (456) | 9.74 |
| Degree | 33.07 (1632) | 33.31 (1556) | 33.09 |
| *Total* | *100 (4935)* | *100 (4671)* | *100 (4994)* |
| *Missing (N)* | *59* | *323* | *N/A* |
| **Household income** |  |  |  |
| <$500 | 12.74 (602) | 12.67 (592) | 12.70 |
| $500-999 | 33.07 (1563) | 33.08 (1545) | 33.12 |
| $1000-1999 | 40.89 (1933) | 40.98 (1914) | 40.91 |
| $2000+ | 13.31 (629) | 13.27 (620) | 13.27 |
| *Total* | *100 (4727)* | *100 (4671)* | *100 (4994)* |
| *Missing (N)* | *267* | *323* | *N/A* |
